# Using flexible methods to determine risk factors for ventilator-associated pneumonia in the Netherlands

**DOI:** 10.1371/journal.pone.0218372

**Published:** 2019-06-20

**Authors:** Tjallie I. I. van der Kooi, Hendriek Boshuizen, Jan C. Wille, Sabine C. de Greeff, Jaap T. van Dissel, Annelot F. Schoffelen, Rolina D. van Gaalen

**Affiliations:** 1 Department of Healthcare-associated Infections and Antimicrobial Resistance, Epidemiology and Surveillance, Centre for Infectious Disease Control, National Institute for Public Health and the Environment, Bilthoven, the Netherlands; 2 Department of Statistics, Informatics and Mathematical Modeling, Centre for Nutrition, Prevention and Health Services, National Institute for Public Health and the Environment, Bilthoven, the Netherlands; 3 Center for Infectious Disease Control, National Institute for Public Health and the Environment, Bilthoven, the Netherlands; 4 Department of Infectious Disease Modelling, Epidemiology and Surveillance, Center for Infectious Disease Control, National Institute for Public Health and the Environment, Bilthoven, the Netherlands; University of Notre Dame Australia, AUSTRALIA

## Abstract

Seven hospitals participated in the Dutch national surveillance for ventilator-associated pneumonia (VAP) and its risk factors. We analysed time-independent and time-dependent risk factors for VAP using the standard Cox regression and the flexible Weighted Cumulative Effects method (WCE) that evaluates both current and past exposures. The prospective surveillance of intensive care patients aged ≥16 years and ventilated ≥48 hours resulted in the inclusion of 940 primary ventilation periods, comprising 7872 ventilation days. The average VAP incidence density was 10.3/1000 ventilation days. Independent risk factors were age (16–40 years at increased risk: HR 2.42 95% confidence interval 1.07–5.50), COPD (HR 0.19 [0.04–0.78]), current sedation score (higher scores at increased risk), current selective oropharyngeal decontamination (HR 0.19 [0.04–0.91]), jet nebulizer (WCE, decreased risk), intravenous antibiotics for selective decontamination of the digestive tract (ivSDD, WCE, decreased risk), and intravenous antibiotics not for SDD (WCE, decreased risk). The protective effect of ivSDD was afforded for 24 days with a delay of 3 days. For some time-dependent variables, the WCE model was preferable over standard Cox proportional hazard regression. The WCE method can furthermore increase insight into the active time frame and possible delay herein of a time-dependent risk factor.

## Introduction

Invasively ventilated patients are at an increased risk of acquiring pneumonia, leading to longer hospital stays and increased mortality. Ventilator-associated pneumonia (VAP) rates ranging from 2 to over 20/1000 ventilation days have been reported[1–3] with attributable mortality of 1–13%, depending on the method used and patient specialty[4–7]. Cassini et al estimated that healthcare-associated pneumonia, including VAP, leads to a burden of 169 (95%CI 149–192) disability-adjusted life years per 100,000 total population, more than any other healthcare-associated infection[8]. Several patient and treatment characteristics have been demonstrated to be associated with the risk to develop VAP, such as Glasgow coma scale, Apache II score, intubation site, length of hospital/ICU stay before ventilation, neutropenia, stress ulcer prophylaxis, corticosteroids, systemic antibiotics and enteral feeding[9–18].

Some of these patient and treatment characteristics are time-dependent. Longer exposure or treatment will modify the (cumulative) risk, but it is less clear and often not evaluated how the association of these time-dependent risks develop during and following exposure[9, 10, 12, 16, 17, 19–21]. In this manuscript we present the VAP surveillance results and evaluate the risk factors. For the time-dependent risk factors we use both standard Cox regression and the flexible Weighted Cumulative Effects (WCE) approach that evaluates both current and past exposures[22]. The WCE approach allows estimation of the timeframe during which a risk factor is (still) relevant.

## Material and methods

### Study setting and set up

PREZIES (Prevention of HAI through Surveillance) is the Dutch national surveillance network for healthcare associated infections, hosted by the National Institute for Public Health and the Environment (RIVM), in which hospitals participate voluntarily. Since 1996, PREZIES offers Dutch hospitals the possibility to participate in the surveillance of hospital-acquired infections with attendant benchmarks. The VAP surveillance module was offered from January 2004 – December 2011.

**Fig 1:**
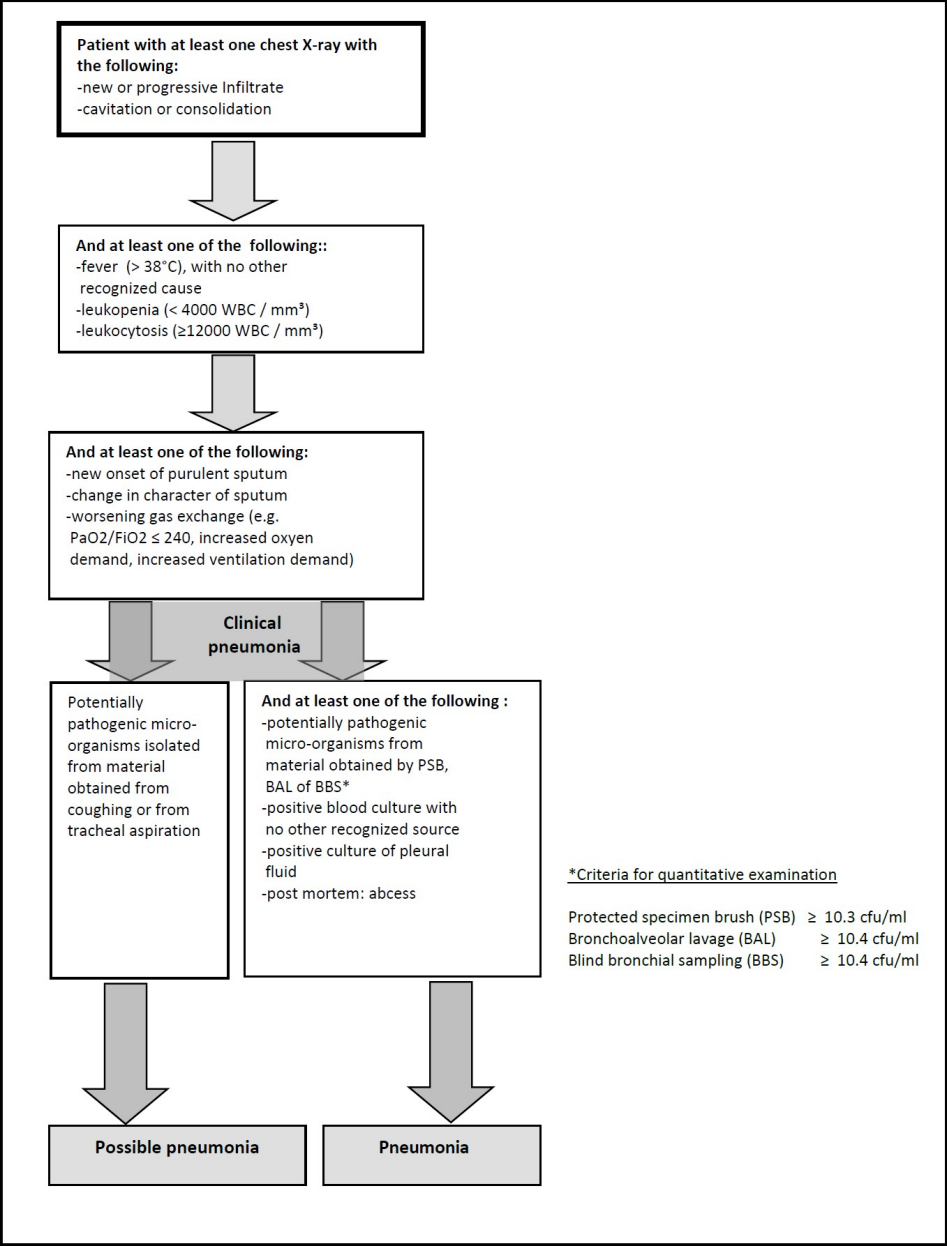
Diagnostic diagram for ventilator-associated pneumonia, based on the definitions of the former Centers for Disease Control and Prevention/National Nosocomial Infections Surveillance system, currently the National Healthcare Safety Network.

The VAP surveillance protocol was developed by a working group of relevant professionals (intensivist, infectious disease specialist, pulmonologist, anaesthesiologist, epidemiologist and infection control professional) and was based on international literature, the preceding ICU-surveillance conducted by PREZIES [23] and a pilot study. The VAP definition used (Fig 1) was a simplified version of that used by the former Centers for Disease Control and Prevention/National Nosocomial Infections Surveillance system, currently the National Healthcare Safety Network definitions. In Dutch clinical practice, a VAP diagnosis is most often based on positive cultures of tracheal aspirates, in combination with clinical symptoms. With a negative culture, the diagnosis of a clinical pneumonia was still possible.

All patients ventilated invasively for two days (48 hours) or more, aged 16 years or older, and present in the ICU were included in the study. Successive ventilation periods of at least two days were recorded until a VAP occurred or until the end of follow-up, 28 days after the start of each ventilation period. Each ICU admission was assigned a unique identifier that could not be used to link admissions at the patient level. Therefore, patients admitted more than once to the ICU were included as separate patients (termed “admissions”). For this manuscript, we considered only data from the first ventilation period of each admission. Data were recorded prospectively, with infection control professionals checking the patients and patient records in the ICU on average twice a week. Suspected VAPs were usually discussed with a dedicated radiologist or intensivist.

- Per admission were recorded: sex, age, admission and discharge dates of hospital and ICU, Apache II score, specialism, type of ICU and reason for end of follow-up.
- Per ventilation period were recorded: start and end date, intubation site, intubation department, stress ulcer prophylaxis, post-surgical ventilation, inhalation trauma, COPD, corticosteroid use (daily dose > 10 mg prednisone) and neutropenia (< 500 granulocytes).
- Per ventilation day were recorded: sedation score (Ramsay score[24] (see Table A in S1 File), feeding mode (>12 hours), oropharyngeal prophylaxis (SOD), intestinal prophylaxis, systemic antibiotics for selective digestive tract decontamination (ivSDD), systemic antibiotics (therapeutic, not for ivSDD) (ivAB), inhalation therapy (metered dose inhaler (MDI), jet nebulizer or none).
- Per infection were recorded: infection date, infection criteria and a maximum of three micro-organisms. When a clinical or possible pneumonia was later followed by respectively a possible and/or (‘confirmed’) pneumonia (for the same infectious episode), the latter were recorded as final diagnosis. In this paper, we do not distinguish between these diagnostic categories.

According to Dutch legislation, written consent from each individual patient was not required because the data from the PREZIES network is anonymized and was collected as a legal task of the National Institute for Public Health and the Environment.

### Statistical analysis

Data are expressed as median (interquartile range (IQR)) and absolute and relative frequencies, as appropriate. A non-parametric test (Kruskal-Wallis) was used to compare median ventilation durations.

We first calculated cause-specific hazards using univariate Cox regression models, adjusted for hospital as a fixed effect. Time-fixed, non-linear, or continuous covariates as well as duration of participation were modelled categorically in separate models using dummy variables. Separately for each time-dependent covariate, we fitted four regression models to determine if, and in which form, the covariate should be included in a multivariate analysis. The four models included three Cox models (current effects and delayed effects of one or two days) and one flexible Cox model using the WCE approach[22]. The WCE approach estimates not only the current effect of a covariate, but also the cumulative delayed effects of past exposures, and provides the timeframe for which a covariate is significantly associated with the studied event. See Box 1 for more details[25]. We assessed which of these four models fitted best using the Akaike information criteria (AIC)[26]. A difference of less than four suggested that the models fit the data equally well, a difference between four and ten suggested a slight difference, and a difference greater than ten suggested a major difference in model fit[27]. We chose the WCE model as the best-fitting model when it yielded a slight or major improvement in model fit to all of the three non-cumulative models.

**Box 1. The WCE method in more detail**

The WCE model uses the Cox PH framework and time-varying covariates to generate, for each covariate, a function that describes the delayed and immediate effect of (past) exposures/levels on the outcome[22].The WCE model requires as input (1) the time-window in which past exposures are considered to have an impact on the risk of the outcome, (2) a pre-specified number of internal knots, which determine the flexibility of the cubic B-spline, and (3) whether the impact of past exposures reaches zero at the earliest point of the time-window (i.e., constraining the effect of the covariate to the null at that point). When insufficient prior information is available to make an informed choice on these inputs, as in our case, the data may be used to determine which inputs provide the best model fit. The approach used to select the optimal WCE model for each factor involved fitting multiple WCE models using all possible time-windows (up to 2, 3, 4, …, 28 days back); 1, 2 or 3 internal knots; and with the effect of the exposure at the most historical time point included in the time-window either unconstrained or constrained to the null.

From these 162 (27 x 3 x 2) models, the best-fitting WCE model for each factor was selected as the WCE model with the lowest Akaike information criteria (AIC). Since we selected the best of multiple models, p-values for WCE univariate models are likely to be artificially low. For each factor, we therefore simulated 1000 datasets in which there was no association, keeping the exposure patterns and outcome times consistent with the original dataset. For each of the 1000 simulated datasets, we ran the same set of WCE models (with alternative time-windows, numbers of internal knots, and weight-function constraints), and selected the best-fitting model using the AIC, as above. The distribution of the 1000 p-values was plotted and the proportion of the 1000 p-values that were smaller than the p-value of the optimal WCE model was recorded as the p-value corrected for multiple testing[25].

All risk factors with p-value<0.2 in the univariate analysis were included in the initial multivariate Cox PH model, using the best-fitting univariate models. The final multivariate model was selected manually by backward selection using the likelihood ratio test. At each iteration, we removed from the model the variable that was associated with the highest p-value>0.05, except when the 95% CI for that variable did not include the null.

The WCE model requires complete data for the entire follow-up of each admission. Since some *time-dependent* data were missing for only 32 ventilation days (0.4%) in 24 admissions (2.6%), we used the last observation carried forward approach to fill in these values for missing days in our regression analyses. The sensitivity of our results to this approach for completing missing data was assessed by removing admissions with missing time-varying data from the dataset and rerunning the multivariate model. Admissions with data missing on *time-fixed* patient or ventilation period characteristics were excluded from the specific analysis.

Analyses were done with SAS 9.4 and R 3.4.1 software using the survival and WCE packages[28–30].

## Results

### Participating hospitals

Seven hospitals (9% of all Dutch hospitals) participated for different periods (1–3.5 years) during the eight years of the VAP surveillance module (2004–2011). Four of the seven hospitals were top clinical (“high cure”) hospitals and three were general hospitals. University hospitals did not participate. The nation-wide distribution of the three hospital types is 29 (top clinical), 61 (general) and 10% (academic).

**Fig 2:**
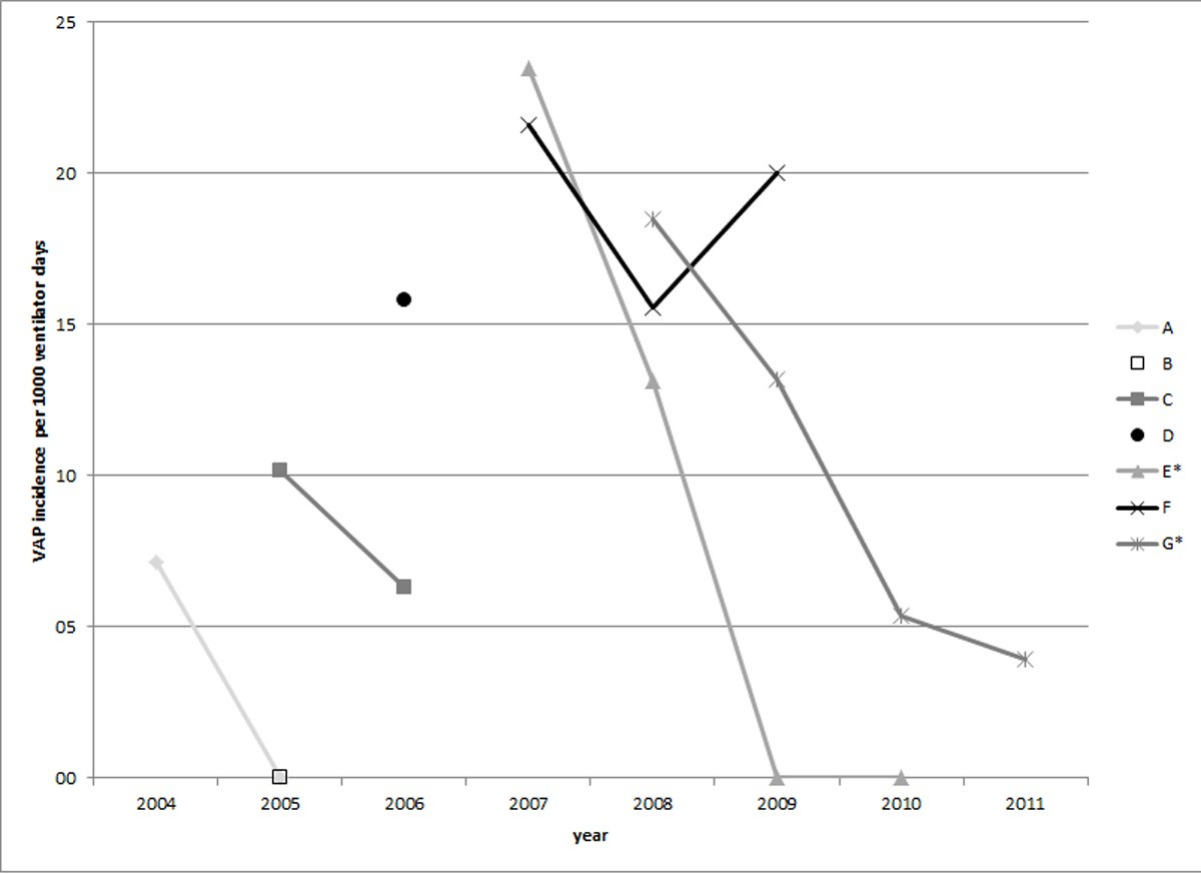
Average yearly incidence density, per 1000 ventilation days, of ventilator-associated pneumonia, per hospital. Hospitals with significant reduction are indicated with an asterisk (*).

In the Netherlands, three ICU levels are discerned, ranging from I (relatively low complex care) to III (high complex care). The participating hospitals had ICU level I (1), II (3) and III (3) (see Table B in S1 File for hospital-specific results). Median ventilation duration differed significantly between hospitals (p<0.0001), ranging from four to eight days. Four of the five hospitals that participated for two years or more demonstrated a reduction in VAP (Fig 2), of which two significantly so.

### Ventilation periods

The surveillance included 940 first ventilation periods of 940 ICU-admissions, all in mixed medical/surgical ICUs, comprising 7872 ventilation days, including all calendar days. The median ventilation duration for all admissions was 6 days (interquartile range (IQR) 4–10 days) (Table B in S1 File); for patients without VAP, 6 days (4–11); and for those that developed VAP, 5 days (3–7) until VAP. During follow-up, the number of patients that were still on the ventilator each day declined exponentially (Figure A in S1 File). Of the 940 admissions, 81 developed a VAP during follow-up (8.6%) and 23 were still on the ventilator on day 28. The average VAP incidence density was 10.3/1000 ventilation days (range between hospitals 0.0 to 20.1).

### Admission characteristics

Our study population included more men (59.3%, median age 68 [IQR 59–76] and APACHE II score 20 [15–26.5]) than women (median age 70 [59–78] and APACHE II score 22 [17–28]), and men developed VAP more often (10.1%; 95% confidence interval (CI) (8.2–12.0%) versus 6.5% (4.9–8.1%)). The median age was 69 (IQR 59–77) years for all admissions and the median Apache II score 21 (IQR 16–27). See Tables 1 and 2 and Table B in S1 File. The median age and Apache II scores were comparable for admissions with and without a VAP.

**Table 1:** Patient and ventilation characteristics and hazard ratios, including 95% confidence intervals and adjusted for hospital, of time-independent covariates.

	Patients	*Perc*. *of patients*	Ventilator days	*Perc*. *of ventilator days*	Number of VAPs	Perc. patients with VAP	Incidence density per 1000 ventilator days	HR (95% CI)	p-value
**Total**	940		7872		81	**8.6%**	**10.3**		
**Sex**									
Man	557	*59*.*3*	4691	59.6	56	**10.1**	**11.9**	**Ref**	
Woman	382	*40*.*6*	3175	40.3	25	**6.5**	**7.9**	**0.64 (0.40–1.02)**	0.06
Missing	1	*0*.*1*	6	0.1	0	**0.0**	**0.0**		
**Age**									
16–40	66	*7*.*0*	409	5.2	13	**19.7**	**31.8**	**3.79 (1.72–8.38)**	0.001
41–60	207	*22*.*0*	1614	20.5	12	**5.8**	**7.4**	**Ref**	
61–80	529	*56*.*3*	4675	59.4	45	**8.5**	**9.6**	**1.38 (0.72–2.61)**	0.33
> 80	138	*14*.*7*	1174	14.9	11	**8.0**	**9.4**	**1.60 (0.68–3.74)**	0.28
**Apache II score**									
≤ 10	143	*15*.*2*	1248	15.9	10	**7.0**	**8.0**	**Ref**	
11–20	339	*36*.*1*	2850	36.2	26	**7.7**	**9.1**	**0.61 (0.26–1.41)**	0.25
21–30	330	*35*.*1*	2790	35.4	34	**10.3**	**12.2**	**0.82 (0.36–1.87)**	0.63
> 30	128	*13*.*6*	984	12.5	11	**8.6**	**11.2**	**0.95 (0.36–2.51)**	0.92
**Specialty**									
Abdominal surgery	359	*38*.*2*	3443	43.7	34	**9.5**	**9.9**	**Ref**	
Cardiology	92	*9*.*8*	515	6.5	5	**5.4**	**9.7**	**1.30 (0.50–3.38)**	0.60
Cardiothoracic surgery	37	*3*.*9*	226	2.9	0	**0.0**	**0.0**	**-**	
Internal medicine	150	*16*.*0*	1418	18.0	7	**4.7**	**4.9**	**0.60 (0.28–1.31)**	0.20
Neurology	56	*6*.*0*	369	4.7	5	**8.9**	**13.6**	**1.33 (0.51–3.44)**	0.56
Neurosurgery	53	*5*.*6*	323	4.1	10	**18.9**	**31.0**	**1.66 (0.76–3.65)**	0.20
Other surgery	93	*9*.*9*	790	10.0	10	**10.8**	**12.7**	**1.10 (0.53–2.28)**	0.80
Pulmonology	47	*5*.*0*	314	4.0	1	**2.1**	**3.2**	**0.31 (0.04–2.26)**	0.25
Traumatology	30	*3*.*2*	316	4.0	8	**26.7**	**25.3**	**1.92 (0.81–4.53)**	0.14
Other specialties	21	*2*.*2*	147	1.9	1	**4.8**	**6.8**	**0.56 (0.08–4.12)**	0.57
Missing	2	*0*.*2*	11	0.1	0	**0.0**	**0.0**	**-**	
**LOS (before ventilation)**									
0 days	322	*34*.*3*	2553	32.4	34	**10.6**	**13.3**	**Ref**	
1	168	*17*.*9*	1446	18.4	17	**10.1**	**11.8**	**0.98 (0.54–1.78)**	0.96
2	94	*10*.*0*	791	10.0	10	**10.6**	**12.6**	**0.99 (0.49–2.01)**	0.97
3–5	132	*14*.*0*	1175	14.9	6	**4.5**	**5.1**	**0.38 (0.16–0.92)**	0.03
6–10	104	*11*.*1*	834	10.6	7	**6.7**	**8.4**	**0.61 (0.27–1.39)**	0.24
>10	120	*12*.*8*	1073	13.6	7	**5.8**	**6.5**	**0.55 (0.25–1.18)**	0.13
**LOS_IC (before ventilation)**									
0 days	746	*79*.*4*	5953	75.6	64	**8.6**	**10.8**	**Ref**	
1	113	*12*.*0*	1109	14.1	12	**10.6**	**10.8**	**1.02 (0.55–1.91)**	0.94
2–5	55	*5*.*9*	558	7.1	3	**5.5**	**5.4**	**0.43 (0.14–1.38)**	0.16
>5	26	*2*.*8*	252	3.2	2	**7.7**	**7.9**	**0.63 (0.20–2.02)**	0.44
**COPD**									
No	808	*86*.*0*	6786	86.2	79	**9.8**	**11.6**	**Ref**	
Yes	131	*13*.*9*	1079	13.7	2	**1.5**	**1.9**	**0.15 (0.04–0.60)**	0.008
Missing	1	*0*.*1*	7	0.1	0	**0.0**	**0.0**		
**Postsurgical ventilation**									
No	468	*49*.*8*	3612	45.9	**35**	**7.5**	**9.7**	**Ref**	
Yes, abdominal surgery	338	*36*.*0*	3231	41.0	**33**	**9.8**	**10.2**	**0.97 (0.60–1.56)**	0.88
Yes, thoracical surgery	30	*3*.*2*	178	2.3	**1**	**3.3**	**5.6**	**2.31 (0.32–16.9)**	0.41
Yes, other surgery	104	*11*.*1*	851	10.8	**12**	**11.5**	**14.1**	**0.88 (0.44–1.74)**	0.71
**Intubation site**									
Oral	918	*97*.*7*	7641	97.1	**79**	**8.6**	**10.3**	**Ref**	
Nasal	6	*0*.*6*	53	0.7	**0**	**0.0**	**0.0**	**0.00 (0.00 –inf)**	0.99
Tracheostoma	14	*1*.*5*	167	2.1	**1**	**7.1**	**6.0**	**0.37 (0.05–2.68)**	0.32
Other	2	*0*.*2*	11	0.1	**1**	**50.0**	**90.9**	**6.73 (0.92–49.1)**	0.06
**Intubation department**									
ICU	417	*44*.*4*	3618	46.0	**25**	**6.0**	**6.9**	**Ref**	
OR	396	*42*.*1*	3505	44.5	40	**10.1**	**11.4**	**1.45 (0.88–2.38)**	0.14
Recovery	5	*0*.*5*	50	0.6	**0**	**0.0**	**0.0**	**0.0 (0.00 –inf)**	1.00
Other	122	*13*.*0*	699	8.9	16	**13.1**	**22.9**	**2.31 (1.19–4.48)**	0.01
**Inhalation trauma**									
No	924	*98*.*3*	7738	98.3	**79**	**8.5**	**10.2**	**1**	
Yes (burns & other)	15	*1*.*6*	131	1.7	**2**	**13.3**	**15.3**	**1.89 (0.44–7.84)**	0.40
burns	1	*0*.*1*	4	0.1	**0**	**0.0**	**0.0**		
other	14	*1*.*5*	127	1.6	**2**	**14.3**	**15.7**		
Missing	1	*0*.*1*	3	<0.1	**0**	**0.0**	**0.0**		
**Stress Ulcer prophylaxis**									
No	346	*36*.*8*	2316	29.4	22	**6.4**	**9.4**	**Ref**	
Protonpump inhibitors	561	*59*.*7*	5296	67.3	56	**10.0**	**10.6**	**1.07 (0.62–1.87)**	0.80
H_2_-antagonists	33	*3*.*5*	260	3.3	3	**9.1**	**11.5**	**1.77 (0.45–6.89)**	0.41
Sucralphate	0	*0*.*0*	0	0.0					
**Corticosteroid use (eq. > 10 mg prednisone)**									
No	489	*52*.*0*	4139	52.6	50	**10.2**	**12.1**	**Ref**	
Yes	451	*48*.*0*	3733	47.4	31	**6.9**	**8.3**	**0.69 (0.43–1.12)**	0.13
**Neutropenia**									
No	905	*96*.*3*	7535	95.7	77	**8.5**	**10.2**	**Ref**	
Yes	32	*3*.*4*	322	4.1	3	**9.4**	**9.3**	**0.71 (0.22–2.26)**	0.59
Missing	3	*0*.*3*	15	0.2	1	**33.3**	**66.7**		
**Participation year**									
1	370	*39*.*4*	2996	38.1	35	**9.5**	**11.7**	**Ref**	
2	284	*30*.*2*	2435	30.9	26	**9.2**	**10.7**	**0.70 (0.41–1.19)**	0.19
3	203	*21*.*6*	1687	21.4	18	**8.9**	**10.7**	**0.52 (0.29–0.95)**	0.03
4	83	*8*.*8*	754	9.6	2	**2.4**	**2.7**	**0.17 (0.04–0.72)**	0.02
Ventilation duration*									
3 calendar days	153	*16*.*3*	2820	35.8	22	**14.4**	**7.8**		
4–5 days	256	*27*.*2*	1429	18.2	21	**8.2**	**14.7**		
6–10 days	299	*31*.*8*	1897	24.1	31	**10.4**	**16.3**		
> 10 days	232	*24*.*7*	1726	21.9	7	**3.0**	**4.1**		

* Calculated according to Mc Laws.

**Table 2:** Ventilation day characteristics and hazard ratios for univariate models of time-dependent covariates.

			Best-fitting *** non-cumulative exposure-risk model (current effect of the current or delayed value of the time-dependent variable)					WCE exposure-risk model (current effect of the current and past values of the time-varying variable for the best-fitting model)				Exposure-risk model selected **** for inclusion in final model
	Original ventilator days (%) *	LOCF ventilator days (%) **	Optimal model	HR	95% CI	p-value	AIC	Relevant exposure window & number of knots for best-fitting model	HR & 95%CI	Adjusted p-value (multiple testing)*****	AIC	
**Sedation score**			Current				977	22 days	Fig 3A	0.162	980	Current
1	737 (9.4)	741 (9.4)		0.07	(0.01, 0.52)	0.010		1 knot for all 5				
2	1095 (13.9)	1095 (13.9)		0.44	(0.19, 1.01)	0.051						
3	944 (12.0)	944 (12.0)		0.39	(0.18, 0.87)	0.020						
4	1207 (15.3)	1208 (15.3)		0.41	(0.20, 0.83)	0.013						
5	1929 (24.5)	1932 (24.5)		0.67	(0.38, 1.19)	0.175						
6	1950 (24.8)	1952 (24.8)		Ref								
missing	10 (0.1)	0 (0.0)										
**Feeding mode**			2-day delay				969	8 days	Fig 3G	0.929	972	2-day delay
No feeding	592 (7.5)	592 (7.5)		2.26	(1.06, 4.78)	0.034		1 knot for both		(combined)		
Parenteral	1238 (15.7)	1248 (15.9)		Ref								
Enteral & Both	6001 (76.2)	6032 (76.6)		0.88	(0.46, 1.67)	0.692						
missing	41 (0.5)	0 (0.0)										
**Inhalation therapy**			2-day delay				987	28 days	Fig 3F	0.048	975	WCE
None	2694 (34.2)	2695 (34.3)		Ref				3 knots for both		(combined)		
Jet nebulizer	1506 (19.1)	1506 (19.1)		1.44	(0.48, 4.27)	0.513						
Metered dose inhaler	3671 (46.6)	3671 (46.6)		0.62	(0.35, 1.11)	0.107						
missing	1(<0.1)	0 (0.0)										
**Systemic AB (not ivSDD)**			2-day delay				983	5 days	Fig 3C	0	961	WCE
Yes	5226 (66.4)	1469 (18.7)		0.56	(0.36, 0.89)	0.015		1 knot				
No	2645 (33.6)	6403 (81.3)		Ref								
missing	1 (0.01)	0 (0.0)										
**ivSDD**			2-day delay				986	28 days	Fig 3B	0.003	973	WCE
Yes	1160 (14.7)	1160 (14.7)		0.43	(0.15, 1.26)	0.123		1 knot				
No	6710 (85.2)	6712 (85.3)		Ref								
missing	2 (0.03)	0 (0.0)										
**Intestinal prophylaxis**			Current				970	12 days	Fig 3E	0	965	WCE
Yes	2395 (30.4)	2395 (30.4)		0.12	(0.04, 0.41)	0.0007		2 knots				
No	5475 (69.6)	5477 (69.6)		Ref								
missing	2 (0.03)	0 (0.0)										
**Oropharyngeal prophylaxis**			Current				967	21 days	Fig 3D	0	971	Current
Yes	4783 (60.8)	4784 (60.8)		0.14	(0.05, 0.38)	0.0001		1 knot				
No	3087 (39.2)	3088 (39.2)		Ref				(unconstrained)				
missing	2 (0.03)	0 (0.0)										

* The total number of ventilation days was 7872.

** The number of ventilation days after reducing the numbers of missings for time-dependent covariates with the ‘last observation carried forward (LOCF) approach

*** Selected as the model with the lowest AIC among the current exposure-risk, 1-day delay exposure-risk, and 2-day delay exposure-risk models in Table C in S1 File.

**** Preference given to a non-cumulative model. WCE model selected if the WCE model had an AIC that was 4 units lower than that of the best-fitting non-cumulative model.

***** The p-value was estimated using 1000 bootstrapped data sets to account for multiple testing when selecting the best-fitting WCE model.

The routines and regimens offered to patients differed between hospitals. Apart from one hospital, hospitals preferred either MDI or nebulizers, four of them exclusively. IvSDD was used in three hospitals, intestinal prophylaxis in three, and SOD in five (see Figure B in S1 File). In hospital G, SOD was given for one year, followed by a complete SDD regimen (oropharyngeal and intestinal prophylaxis and four days ivSDD), as part of a study. In hospital E, SOD was initially given occasionally and subsequently extended to all admissions.

### Univariate results

Of the time-independent covariates, age, length of hospital stay before start of the ventilation, COPD, intubation department, and duration of participation with the surveillance (in years) were significantly associated with VAP (Table 1). All time-dependent variables were significantly associated with the risk of acquiring VAP except for type of inhalation therapy (Table 2).

**Fig 3:**
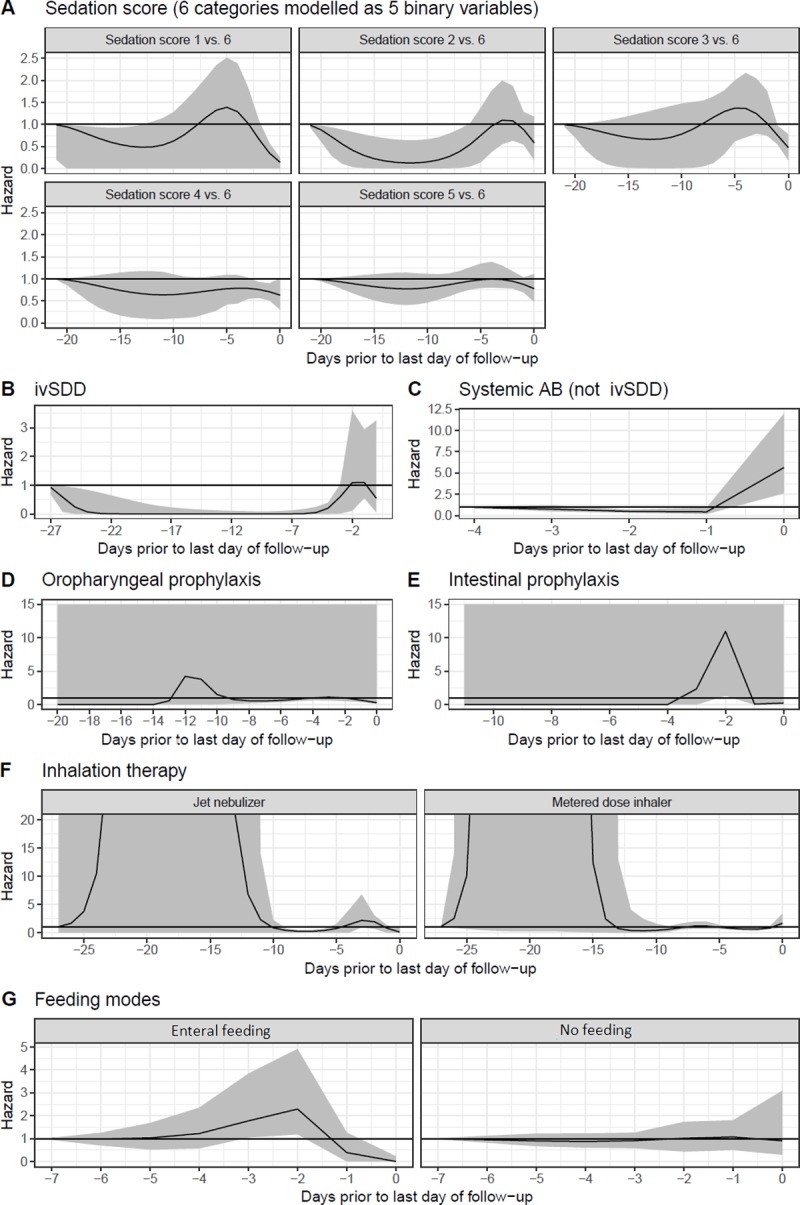
Daily hazards of time-dependent covariates, in univariate models. The curve shows the estimated risk attributed to exposures on each day prior to the last day of follow-up (i.e., the event date or the censoring date) and the grey ribbon shows the 95% confidence interval. A value of one indicates no effect of the exposure at that time. At times where the grey ribbon includes one, the effect is considered to be statistically insignificant.

Fig 3 shows the WCE results for the univariate analyses. The overall hazard ratio of specific exposure patterns compared with uniformly non-exposure can be calculated by multiplying the hazard ratios for all days where, for example, systemic antibiotics (Fig 3C) were administered. Suppose a patient was ventilated for 6 days and treated with ivAB on the first four ventilation days (day -5 to -2), but not on the last two ventilation days (day -1 and 0). The hazard ratio for this patient on the present day (day 0) is a multiplication of the hazards on days -5, -4, -3, and -2. Note that the hazard ratio before day -4 in Fig 3C is assumed to be 1. The WCE model for ivAB suggests a harmful effect of antibiotics taken on the present day (day 0), which is probably a result of reverse causation since system antibiotics were likely taken on the day of a VAP to *treat* the pneumonia. Further, the narrower confidence intervals shown at the left (e.g. Fig 3A) may be seen as counterintuitive since the number of patients declines with increasing follow-up duration (Figure A in S1 File), but are an artefact of the analysis since the best-fitting model for ivAB was one where the hazard ratio was constrained to the null at this point.

### Multivariate results

After backward selection to step-wise exclude covariates from the model, the final multivariate model included age, COPD, current sedation score, current SOD, inhalation therapy (WCE), ivSDD (WCE), and ivAB (WCE) (Table 3).

**Table 3:** Hazard ratios of patient and ventilation characteristics–results of multivariate analysis.

	Hazard ratio (95% CI)	p-value
**Age**		
16–40	2.42 (1.07, 5.50)	0.036
40–60	Ref	
60–80	1.21 (0.62, 2.34)	0.567
> 80	1.58 (0.66, 3.75)	0.305
**COPD**	0.19 (0.04, 0.78)	0.003
**Sedation score, per day (current)**		
1	0.08 (0.01, 0.58)	< 0.001
2	0.67 (0.30, 1.54)	0.335
3	0.46 (0.20, 1.03)	0.048
4	0.43 (0.21, 0.90)	0.062
5	0.77 (0.43, 1.39)	0.388
6	Ref	
**Oropharyngeal prophylaxis (current)**	0.19 (0.04, 0.91)	0.017
**Intravenous antibiotics for SDD (ivSDD)—**(WCE)	See Fig 4A	0.062
**Other systemic AB (not for ivSDD)—**(WCE)	See Fig 4B	< 0.001
**Inhalation therapy—**(WCE)	See Fig 4C	0.009
• Jet nebulizer (compared to no inhalation therapy) • Metered dose inhaler (MDI) (compared to no inhalation therapy)		

**Fig 4:**
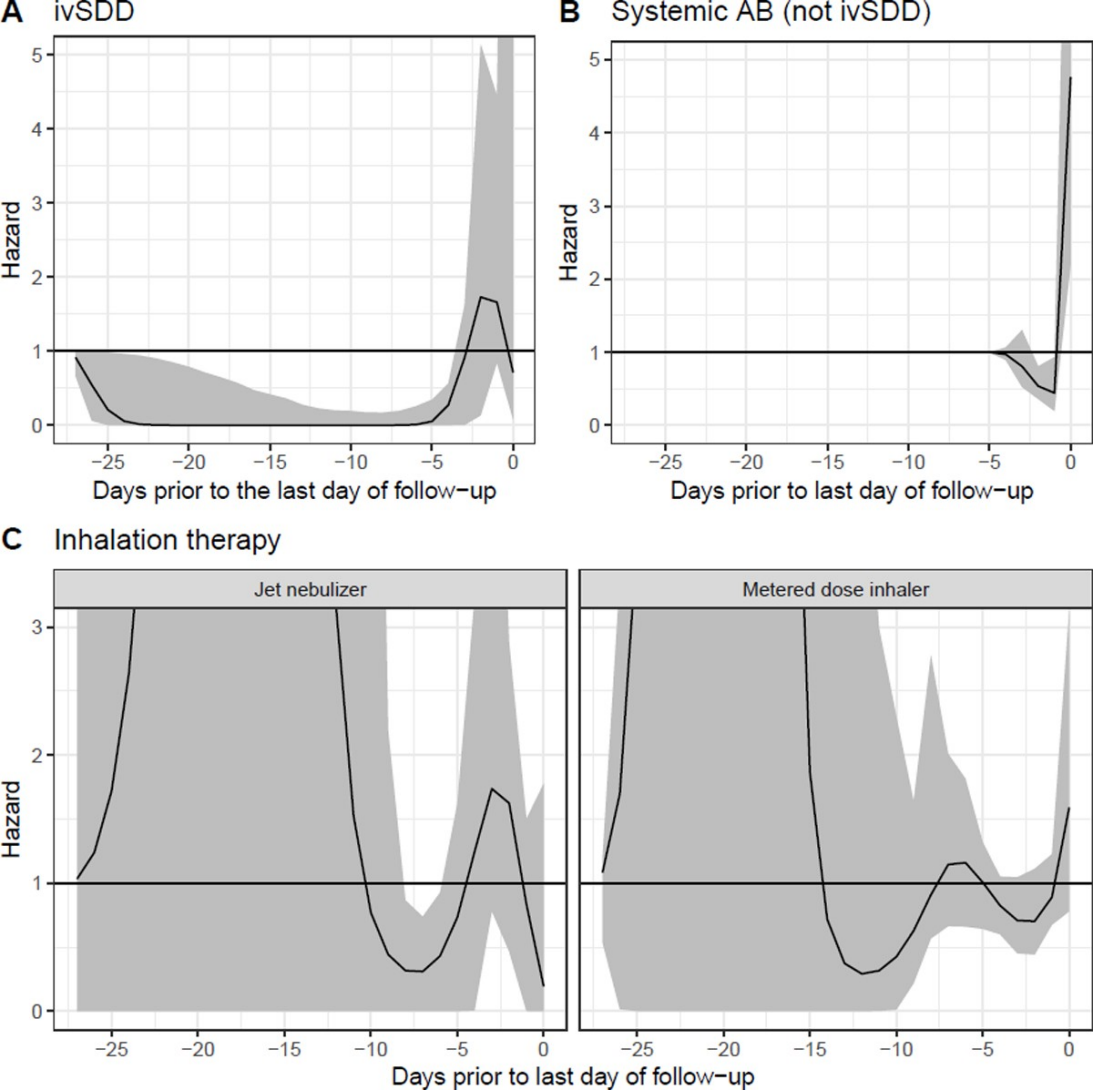
**Daily hazard ratios attributed to past values of the covariates assessed with a weighted cumulative effects approach in the multivariate Cox regression model: (A) ivSDD, (B) ivAB, (C) Jet nebulizer (compared to no inhalation therapy) and metered dose inhalers (MDI) (compared to no inhalation therapy)**. The curve shows the estimated risk attributed to exposures on each day prior to the last day of follow-up (i.e., the event date or the censoring date) and the grey ribbon shows the 95% confidence interval. A value of one indicates no effect of the exposure at that time. At times where the grey ribbon includes one, the effect is considered to be statistically insignificant.

The model suggests that, compared to patients between 40 and 60 years of age, patients ≤ 40 had an over two-fold higher risk of developing VAP. Patients were also at an increased risk when their sedation scores were high. Patients who were on SOD, ivSDD or ivAB and patients with COPD had a lower VAP risk. The model further demonstrates a delayed protective effect of ivSDD by three days. This protective effect is afforded for 24 days (up to 27 days back) (Fig 4A). IvAB was protective for two days, with the protection being afforded after a delay of one day. The effect from inhalation therapy was minimal, with jet nebulizers showing a delayed protective effect for days 6 to 8 back. Our results were not sensitive to using the last observation carry forward approach for completing missing data. Similar results were obtained when we removed admissions with missing time-varying data from the dataset and reran the multivariate model.

## Discussion

In this manuscript, we presented data generated by the VAP surveillance module of the Dutch PREZIES network. The VAP rates reported by the hospitals participating in the surveillance (0–20.1/1000 ventilation days) are in the expected range[1–3]. The observed variation between hospitals could be partly due to the inter-observer reliability of a VAP diagnosis, which is known to be low[31, 32]. However, this applies less to intrahospital comparisons where only one intensivist or radiologist was usually dedicated to surveillance. Four of the five hospitals that participated ≥2 years demonstrated a reduction in VAP, two of which significantly so. Apart from a possible effect of the surveillance itself[33], various changes during the follow-up could have caused this reduction. Several hospitals (C-G) implemented interventions (SOD and/or ivSDD, Evac cuffs, closed suctioning system, VAP bundle), sometimes temporarily. Although the increase in VAP incidence in hospital F was duly investigated at that time, no cause was found. Hospital A did not introduce any interventions. Hospital B, with zero VAPs, used a complete SDD regimen during the surveillance. Therefore, while hospitals appear to be able to reduce VAP, either by introducing an intervention or by surveillance alone, success does not seem to be guaranteed.

The observed effects of patient and treatment characteristics vary among studies[9, 11–18], resulting from differences in the other measured covariates and case mix, and, frequently, low statistical power. In our results, Apache II score, specialty, intubation site, length of hospital/ICU stay before ventilation, postsurgical ventilation, inhalation trauma, stress ulcer prophylaxis, corticosteroids, neutropenia, intestinal prophylaxis, and feeding method were not significantly associated with VAP. Although the overall study population in our study was relatively large, low numbers of patients in certain categories could explain failures to detect associations. In our data, a higher Ramsay score was associated with an increased VAP risk, which corresponds with the increased risk associated with coma or increased Glasgow coma scale[9–12, 16, 17]. Systemic antibiotics (not for ivSDD) appeared to lower the VAP risk, as identified by others[9, 10]. In most analyses where antibiotic use was not analysed as a time-dependent variable, ‘prior antibiotic use’ (or variations) was not found to be associated with VAP[12, 16, 17]. Our results demonstrated that both ivSDD and SOD were associated with a VAP reduction. De Smet et al, performing a multicenter randomized clinical trial, concluded that both SOD and SDD (full regimen) led to a reduction in respiratory tract colonisation with highly resistant microorganisms[21]. As intestinal prophylaxis partly coincided with ivSDD and SOD, we evaluated a model without ivSDD/SOD. Intestinal prophylaxis was not significantly associated with VAP in this model either.

More surprisingly, because unlike other studies where the risk for older patients was similar or increased[3, 11, 17, 18] younger patients (16–40 years) appeared to be at increased risk. This may have resulted from an overrepresentation of young patients in neurosurgery and traumatology, both specialties with high VAP rates. This association remained borderline significant when adjusted for specialty and Apache II score or when these two specialties were excluded from the analysis. When excluding one center, where 11 of the 32 patients aged 16–40 developed pneumonia, the association was still present (HR 3.2 (0.49–21.2), although not significant anymore. While COPD is generally found to be either unassociated with VAP[3, 16], or to increase the VAP risk[11, 17], here COPD appeared to be associated with a lower risk. Our model did not detect a significant interaction with systemic antibiotics. In previous studies, COPD patients had longer ventilation durations relative to patients without COPD[3, 34]. In our study, the first ventilation periods for patients with and without COPD were similar in duration (average of 8.2 and 8.4 days, respectively), as was the total ventilated duration (9.3 and 8.7 days) and the proportion of ventilation periods lasting 28 days(1.4 and 2.8%, respectively), suggesting that the similarities in ventilated duration are not subject to censoring bias. Furthermore, mortality data, available from five hospitals, showed that mortality was comparable (40.4% and 38.5%, respectively). The lower risk in our study may reflect differences in criteria for COPD diagnosis[35], but also improved care. For example, Funk analysed the outcomes of COPD patients admitted in ICU, for the period 1998–2008, and found that risk-adjusted mortality had improved[36].

The use of jet nebulizers demonstrated a slightly reduced risk compared to no inhalation therapy, whereas MDI did not. Appropriate delivery of medication is reported to be more challenging with MDI than with jet nebulizers. In one study no difference in VAP risk was found between both methods, but the population size was small[37].

For the daily measured ivAB, ivSDD, and inhalation therapy, the WCE approach explained the associated VAP risk better than the standard Cox regression analysis. Additionally, this method provides insight into the cumulative effect over time and the relevant retrospective timeframe in which exposures are associated with a VAP. IvSDD affected this hazard for 24 days. It must be noted that in one hospital many patients received ivSDD throughout the entire ventilation period instead of the currently recommended first four days only. Although differences in the AIC were usually small (Table C in S1 File) the WCE approach identified ivSDD as an independent factor affecting the risk to develop VAP whereas standard Cox regression of current values or of one or two days earlier did not. The WCE approach is worth considering in future analysis of time-dependent risk factors of VAP, and for other device-associated infections.

Our study has some limitations. First, while the relatively large study population and the availability of daily data on time-varying risk factors represent strengths, our study made use of surveillance data and therefore missed more detailed clinical data, e.g. type of ivAB or the presence of sepsis, that could also be associated with VAP and affect estimated associations for other factors. Second, hospital-level treatment preferences resulted in low variability within individual hospitals, reducing the power to detect associations on these variables. Third, our results may not be generalizable to other settings. VAP in the Netherlands is usually diagnosed and treated based upon clinical features and/or tracheal aspirate cultures. Therefore, some risk factors may differ when VAP is diagnosed in a more invasive way. Top clinical hospitals were overrepresented in the sample of participating hospitals, whereas academic hospitals did not participate. Along with the variation in VAP rates observed this implies the average VAP incidence density may not be representative of all hospitals. Fourth, we could not distinguish between new and recurrent ICU admissions, which may have led to some overrepresentation of complex patients. Fifth, including only the first ventilation episode may have led to some bias in the assessment of risk factors. Lastly, in-house infection control professionals collected the data. Apart from the low inter-observer reliability of diagnosing VAP[31, 32], this may have led to differences in the application of the surveillance protocol. We aimed to minimize this possible bias by arranging meetings for the involved professionals to discuss data collection and infection criteria.

Surveillance results are limited for clinical use or pathophysiological insight but the data of Dutch ICUs participating in the VAP surveillance system revealed risk factors on both patient (age, sedation score) and treatment level (SDD, oropharyngeal prophylaxis, other antibiotics, nebulizer type) that can be useful for case mix adjustment and evaluation of VAP prevention strategies. The introduction of SDD or oropharyngeal prophylaxis was associated with low or zero VAP incidences. Surprisingly, COPD was associated with a reduced VAP risk, which merits further evaluation. For some time-dependent covariates, the WCE approach was preferable over standard Cox proportional hazard regression and additionally provided insight into the relevant retrospective timeframe of past exposures.

## Supporting information

S1 File Supporting Figures and Tables. (Figure A) Survival without VAP and number of patients per day (still) on the ventilator.(Figure B) Hospitals and calendar years where intravenous antibiotics for selective decontamination of the digestive tract (ivSDD), oropharyngeal and intestinal prophylaxis were given. (Table A) Ramsay sedation score. (Table B) Patient and ventilation characteristics of individual hospitals. (Table C) Hazard ratios for univariate models of time-dependent covariates.
(DOCX)
