# Supplementary material for: Using flexible methods to determine risk factors for ventilator-associated pneumonia in the Netherlands
Source: PLoS One. 2019 Jun 20;14(6):e0218372. doi: 10.1371/journal.pone.0218372 (PMC6586305; doi:10.1371/journal.pone.0218372)
Supplement: S1 File — (Figure A) Survival without VAP and number of patients per day (still) on the ventilator.(Figure B) Hospitals and calendar years where intravenous antibiotics for selective decontamination of the digestive tract (ivSDD), oropharyngeal and intestinal prophylaxis were given. (Table A) Ramsay sedation score. (Table B) Patient and ventilation characteristics of individual hospitals. (Table C) Hazard ratios for univariate models of time-dependent covariates. (DOCX) [file pone.0218372.s001.docx]

# S1 Supporting figures and tables

Figure A: Survival without VAP and number of patients per day (still) on the ventilator

Figure B: Hospitals and calendar years where intravenous antibiotics for selective decontamination of the digestive tract (ivSDD), oropharyngeal and intestinal prophylaxis were given.

Table A: Ramsay sedation score

| Awake |
| --- |
| 1. Patient anxious, agitated or restless or both |
| 2. Patient co-operative, orientated and tranquil |
| 3. Patient responds to commands only |
|  |
| Asleep levels depend on the patient’s response to a light glabellar tap or loud auditory stimulus |
| 4. Brisk response |
| 5. Sluggish response |
| 6. No response |

Table B: Patient and ventilation characteristics of individual hospitals.

| **Hospitals** | **Total** | **A** | **B** | **C** | **D** | **E** | **F** | **G** |
| --- | --- | --- | --- | --- | --- | --- | --- | --- |
| ICU level (I low, III highly complex care) |  | II | III | I | II | III | III | II |
| Patients | 940 | 89 | *140* | 61 | 21 | 210 | 265 | 154 |
| % Men* | *59* | *69* | *60* | *67* | *52* | *58* | *58* | *55* |
| Median age (IQR) | 69 (59-77) | 70 (58-79) | 67.5 (58-75) | 71 (60-81) | 68 (56-78) | 73 (63-79) | 63 (51-72) | 71 (63-79) |
| Median Apache II score (IQR) | 21 (16-27) | not recorded | 26 (19-31) | 15 (11-23) | 22 (20-27) | 21 (16-27) | 21 (16-26) | 20 (16-26) |
| % COPD* | *14* | *24* | *0* | *10* | *24* | *20* | *14* | *13* |
| Median ventilation duration (IQR) | 6 (4-10) | 7 (5-12) | 4 (3-6) | 8 (5-19) | 7 (5-13) | 7 (4-12) | 6 (4-9) | 7 (4-12) |
| Median duration of ventilation in case of VAP (IQR) | 5 (3-7) | 12 (7-21) | no VAP | 7.5 (6-10) | 5 (3-5) | 6 (4-8) | 4 (3-7) | 4 (3-9) |

* = Missing for one patient

Table C: Hazard ratios for univariate models of time-dependent covariates

|  |  | **Current exposure-risk model (current effect of the current value of the time-dependent variable)** | | | | **1-day delay exposure-risk model (current effect of the current value of the time-dependent variable)** | | | | **2-day delay exposure-risk model (current effect of the current value of the time-dependent variable)** | | | | **WCE exposure-risk model (current effect of the current and past values of the time-dependent variable for the best-fitting model)** | | | | **Exposure-risk model selected for inclusion in final model** |
| --- | --- | --- | --- | --- | --- | --- | --- | --- | --- | --- | --- | --- | --- | --- | --- | --- | --- | --- |
|  | **LOCF ventilator days (%) *** | **HR** | **95% CI** | **p-value** | **AIC** | **HR** | **95% CI** | **p-value** | **AIC** | **HR** | **95% CI** | **p-value** | **AIC** | **Relevant exposure window & number of knots for best-fitting model** | **HR & 95%CI** | **p-value**** | **AIC** |  |
| **Sedation score** |  |  |  |  | 977 |  |  |  | 983 |  |  |  | 986 | 22 days | Fig 3A | 0.162 | 980 | Current |
| 1 | 741 (9.4) | 0.07 | (0.01, 0.52) | 0.010 |  | 0.11 | (0.01, 0.83) | 0.032 |  | 0.16 | (0.02, 1.22) | 0.077 |  | 1 knot for all 5 |  |  |  |  |
| 2 | 1095 (13.9) | 0.44 | (0.19, 1.01) | 0.051 |  | 0.50 | (0.20, 1.26) | 0.141 |  | 0.47 | (0.16, 1.37) | 0.167 |  |  |  |  |  |  |
| 3 | 944 (12.0) | 0.39 | (0.18, 0.87) | 0.020 |  | 0.31 | (0.12, 0.77) | 0.012 |  | 0.47 | (0.20, 1.14) | 0.094 |  |  |  |  |  |  |
| 4 | 1208 (15.3) | 0.41 | (0.20, 0.83) | 0.013 |  | 0.52 | (0.27, 1.02) | 0.058 |  | 0.39 | (0.18, 0.84) | 0.015 |  |  |  |  |  |  |
| 5 | 1932 (24.5) | 0.67 | (0.38, 1.19) | 0.175 |  | 0.67 | (0.38, 1.18) | 0.167 |  | 0.70 | (0.41, 1.20) | 0.192 |  |  |  |  |  |  |
| 6 | 1952 (24.8) | Ref |  |  |  | Ref |  |  |  | Ref |  |  |  |  |  |  |  |  |
| **Feeding mode** |  |  |  |  | 989 |  |  |  | 990 |  |  |  | 969 | 8 days | Fig 3G | 0.929 | 989 | 2-day delay |
| No feeding | 592 (7.5) | 0.00 | (0.00, inf) | 0.998 |  | 0.46 | (0.06, 3.39) | 0.445 |  | 2.26 | (1.06, 4.78) | 0.034 |  | 1 knot for both |  | (combined) |  |  |
| Parenteral | 1248 (15.9) | Ref |  |  |  | Ref |  |  |  | Ref |  |  |  |  |  |  |  |  |
| Enteral&Both | 6032 (76.6) | 0.92 | (0.51, 1.65) | 0.772 |  | 0.87 | (0.48, 1.58) | 0.651 |  | 0.88 | (0.46, 1.67) | 0.692 |  |  |  |  |  |  |
| **Inhalation therapy** |  |  |  |  | 989 |  |  |  | 989 |  |  |  | 987 | 28 days | Fig 3F | 0.048 | 975 | WCE |
| None | 2695 (34.3) | Ref |  |  |  | Ref |  |  |  | Ref |  |  |  | 3 knots for both |  | (combined) |  |  |
| Nebulizer | 1506 (19.1) | 0.41 | (0.09, 1.98) | 0.269 |  | 0.69 | (0.18, 2.63) | 0.590 |  | 1.44 | (0.48, 4.27) | 0.513 |  |  |  |  |  |  |
| Metered dose inhaler | 3671 (46.6) | 0.94 | (0.55, 1.61) | 0.833 |  | 0.74 | (0.43, 1.23) | 0.288 |  | 0.62 | (0.35, 1.11) | 0.107 |  |  |  |  |  |  |
| **Systemic AB**  **(not ivSDD)** |  |  |  |  | 985 |  |  |  | 986 |  |  |  | 983 | 5 days | Fig 3C | 0 | 961 | WCE |
| Yes | 5227 (66.4) | 1.71 | (0.99, 2.98) | 0.056 |  | 0.67 | (0.42, 1.07) | 0.093 |  | 0.56 | (0.36, 0.89) | 0.015 |  | 1 knot |  |  |  |  |
| No | 2645 (33.6) | Ref |  |  |  | Ref |  |  |  | Ref |  |  |  |  |  |  |  |  |
| **ivSDD** |  |  |  |  | 988 |  |  |  | 987 |  |  |  | 986 | 28 days | Fig 3B | 0.003 | 973 | WCE |
| Yes | 1160 (14.7) | 0.65 | (0.22, 1.92) | 0.44 |  | 0.51 | (0.18, 1.51) | 0.23 |  | 0.43 | (0.15, 1.26) | 0.13 |  | 1 knot |  |  |  |  |
| No | 6712 (85.3) | Ref |  |  |  | Ref |  |  |  | Ref |  |  |  |  |  |  |  |  |
| **Intestinal prophylaxis** |  |  |  |  | 970 |  |  |  | 970 |  |  |  | 975 | 12 day | Fig 3E | 0 | 965 | WCE |
| Yes | 2395 (30.4) | 0.12 | (0.04, 0.41) | 0.0007 |  | 0.13 | (0.04, 0.42) | 0.0008 |  | 0.19 | (0.06, 0.54) | 0.002 |  | 2 knots |  |  |  |  |
| No | 5477 (69.6) | Ref |  |  |  | Ref |  |  |  | Ref |  |  |  |  |  |  |  |  |
| **Oropharyngeal prophylaxis** |  |  |  |  | 967 |  |  |  | 968 |  |  |  | 973 | 21 days | Fig 3D | 0 | 965 | Current |
| Yes | 4784 (60.8) | 0.14 | (0.05, 0.38) | 0.0001 |  | 0.14 | (0.05, 0.39) | 0.0002 |  | 0.20 | (0.08, 0.51) | 0.0006 |  | 1 knot |  |  |  |  |
| No | 3088 (39.2) | Ref |  |  |  | Ref |  |  |  | Ref |  |  |  | (unconstrained) |  |  |  |  |

*The number of ventilation days after reducing the numbers of missings for time-dependent covariates with the ‘last observation carried forward (LOCF) approach’.
The total number of ventilation days was 7872
**The p-value was estimated using 1000 bootstrapped data sets to account for multiple testing when selecting the best-fitting WCE model.
